# Nomenclature

**Published:** 1883-11

**Authors:** C. E. Francis


### Nomenclature.

BY C. E. FRANCIS, D. D. S.

Although in Dental Science, Art, and Literature, great ad. vances have been made, there is yet room for further progress and more extended improvements. In the field of dental literature especially is this the case. The masses who constitute the bulk of our calling are sadly deficient in literary ability, and with some even the study of English grammar seems to have formed no part of the preparation for their field of duties.

Probably no one will claim that a knowledge of the classics or the highest order of education is indispensable to the dentist, or that it will materially aid him in the ordinary operations performed at his chair; yet, refinement, culture, and a well-stored intellect are requirements of great value to even those who are endowed with natural mechanical tact.

The application of the term "Doctor" implies a superior grade of erudition, and this fact has been ever recognized in all communities.

It would seem strange, indeed, to find an individual to whose signature was appended the letters L. L. D., D. D., or Ph. D., so sadly deficient in matter of education as to be unable to speak or write even in his own native language with any degree of correctness. Then why should not he who parades a degree of M. D., or D. D. S., possess at least an average common school education?

Why should not a class of professional men upon whose acquired knowledge, judgment and advice depend so much that contributes to the health and comfort of communities, and to whose care are entrusted even the lives of the people, possess cultured intellects, and an education upon general matters equal to that of members of other professions?

If a dentist is not strictly a scientist nor his area of learning greatly extended, there seems little excuse for not keeping pace with the gradual advances of his specialty.

What excuse can be offered for persistently clinging to technicalties obsolete, erroneous, or devoid of sense?

The common practice of applying the term "nerve" to the dentinal pulp is a senseless misnomer. To call the first and second molars "six-year-old," and "twelve-year-old" teeth is quite as meaningless. To say "sixth-year-molar" or "twelfth-year-molar" would imply that these teeth made their first appearance at ages thus indicated, therefore are not specially objectionable terms. Aside from correctness of expression, it is certainly more euphonic to say "the superior first molar" than the "upper six-year-old molar."

Why call the cuspids "eye-teeth" and "stomach-teeth?" It is easier to say "superior cuspid" than "upper eye-tooth."

The term "tartar" applied to the various accumulations about the teeth is one of the most incorrect expressions in the dentist's vocabulary, and has no significance there. It is applied alike to the ordinary salivary calculus, the dusky mucoid secretions, and all other extraneous collections whatever their character or composition.

